# Naïve CD4+ T Cell Lymphopenia and Apoptosis in Chronic Hepatitis C Virus Infection Is Driven by the CD31+ Subset and Is Partially Normalized in Direct-Acting Antiviral Treated Persons

**DOI:** 10.3389/fimmu.2021.641230

**Published:** 2021-04-12

**Authors:** Ann W.N. Auma, Carey L. Shive, Alyssa Lange, Sofi Damjanovska, Corinne Kowal, Elizabeth Zebrowski, Pushpa Pandiyan, Brigid Wilson, Robert C. Kalayjian, David H. Canaday, Donald D. Anthony

**Affiliations:** ^1^ Department of Pathology, Case Western Reserve University, Cleveland, OH, United States; ^2^ GRECC, VA Northeast Ohio Healthcare System, Cleveland, OH, United States; ^3^ Department of Medicine, MetroHealth Medical Center, Case Western Reserve University, Cleveland, OH, United States

**Keywords:** hepatitis c virus infection, naïve cd4+ T cells, apoptosis, lymphopenia, direct-acting antiviral

## Abstract

**Background:**

The mechanisms underlying naïve CD4+ lymphopenia during chronic Hepatitis C Virus (HCV) infection are unclear. Whether direct-acting antiviral (DAA) therapy restores peripheral naïve CD4+ T cell numbers and function is unknown.

**Methods:**

We enumerated frequencies and counts of peripheral naïve CD4+, CD4+CD31+ and CD4+CD31- T cells by flow cytometry in a cross sectional analysis comparing chronic HCV infected (n=34), DAA-treated(n=29), and age-range matched controls (n=25), as well as in a longitudinal cohort of HCV DAA treated persons (n=16). The cross-sectional cohort was stratified by cirrhosis state. Cell apoptosis/survival (AnnexinV+7AAD+/BCL-2 labeling) and cell cycle entry (Ki67 expression) of CD31+ and CD31- naïve CD4+ T cells was analyzed directly *ex vivo* and following 3 and 5 days of *in vitro* culture with media, interleukin (IL) -7 or CD3/CD28 activator.

**Results:**

In the cross-sectional cohort, naïve CD4+ proportions were lower in chronic HCV infected persons compared to controls and DAA-treated persons, an effect in part attributed to cirrhosis. Age was associated with naïve cell counts and proportions in HCV infected and treated persons as well. Naïve CD4+ cell proportions negatively correlated with plasma levels of soluble CD14 following therapy in DAA-treated persons. Naïve CD4+ cells from HCV infected persons exhibited greater direct *ex vivo* apoptosis and cell-cycling compared to cells from DAA-treated persons and controls, and this was localized to the CD4+CD31+ subset. On the other hand, no remarkable differences in expression of BCL-2 or IL-7 Receptor (CD127) at baseline or following *in vitro* media or IL7 containing culture were observed. In the longitudinal cohort, naïve CD4+CD31+/CD31- ratio tended to increase 24 weeks after DAA therapy initiation.

**Conclusions:**

Activation and apoptosis of peripheral naïve CD4+CD31+ T cells appear to contribute to naïve CD4+ lymphopenia in chronic HCV infection, and this defect is partially reversible with HCV DAA therapy. Age and cirrhosis -associated naïve CD4+ lymphopenia is present both before and after HCV DAA therapy. These findings have implications for restoration of host immune function after DAA therapy.

## Introduction

Chronic Hepatitis C virus (HCV) infection is associated with impaired immunity against neoantigens contained in vaccines and new infections (1). Immunity to neoantigens is dependent upon naïve CD4+ T cell recognition of a broad antigen repertoire (2–4), a function primarily executed by CD31 expressing naïve CD4+ T cells that have been demonstrated to contain a polyclonal and diverse T cell receptor (TCR) repertoire (5). Indeed, naïve CD4+CD31+ lymphopenia attributed to age-related thymic involution and subsequently diminished thymic output in the elderly was associated with impaired immunity to vaccines and new infections (6, 7). Along these lines, the poor vaccine responses in chronic HCV infected persons (1) may be partly attributable to the naïve CD4+CD31+ lymphopenia observed in this group (8). Furthermore, the naïve CD4+CD31+ T cells are considered to be enriched for recent thymic emigrants (RTEs) and have greater numbers of TRECs (T cell receptor excision circles); deoxyribonucleic acid (DNA) by-products of TCR gene rearrangement (9). Naïve CD4+CD31+ T cell lymphopenia is associated with low TREC numbers in the elderly and both are biomarkers of thymic function (9, 10). Previous studies have used these parameters to demonstrate low thymic function during ageing and chronic HCV infection (5, 11, 12). Whether chronic HCV infection treatment can improve naïve CD4+ cell numbers, particularly during older age, is not clear. Interferon therapy has been observed to lower naïve CD4+CD31+ T cell counts (13), but the impact of direct-acting antiviral (DAA) interferon-free therapy is unknown and thus addressed here.

CD4+CD31- T cells undergo peripheral homeostatic proliferation, resulting in reduction of TRECs (TRECs are non-replicating and therefore diluted with each cellular division) (14), a distinguishing feature of this naïve CD4+ subset (9, 10). Further, CD4+CD31- T cells possess a significantly restricted and oligoclonal TCR repertoire when compared to the CD4+CD31+ T cells due to deletion of the CD4+CD31- T cells that receive insufficient homeostatic signals (including IL-7), with a resultant net loss of those specific TCRs from the naïve T cell pool (5). Similar to the CD4+CD31+ subset, naïve CD4+CD31- lymphopenia is also observed in chronic HCV and HIV infections (12, 15). However, in contrast to CD4+CD31+ T cell numbers that decline with age, the CD4+CD31- T cell numbers are either stable or increase with age (15, 16), suggesting that distinct mechanisms underlie naïve CD4+CD31+ and CD4+CD31- T cell homeostasis. CD31 is also expressed on CD8+ naïve T cells but at significantly higher levels compared to CD4+ naïve T cells (17) and it is not clear whether CD31 is a reliable marker for CD8+ RTEs since the published literature is not robust (12, 18–20). In the present study, we aimed to understand the impact of chronic HCV infection on absolute counts and proportions of naïve CD4+ T cells and corresponding CD31+ and CD31- subsets before and after DAA therapy and in uninfected controls. We also investigated the relationship between age and numbers of each naïve CD4+ T cell subtype and determined whether this relationship differed before or after DAA therapy. Finally, we evaluated direct *ex vivo* cell death and cycling, and *in vitro* response to TCR and interleukin (IL) -7 stimulation to gain insight into mechanisms underlying naïve CD4 lymphopenia.

## Methods

### Study Population

Study participants provided written informed consent under protocols approved by the institutional review boards for human studies at the Cleveland Veterans Affairs Medical Center and University Hospitals of Cleveland. The cross-sectional study cohort included age-range matched uninfected controls (n=34), chronic HCV infected (n=29) and HCV DAA-treated (n=25, 1-5 years post-therapy start with successful therapy outcome/sustained virologic response (SVR)) participant groups. The longitudinal study cohort included chronic HCV infected persons scheduled for initiation of HCV DAA therapy and were followed up at weeks 0 (Start), 4, 8, and 24 following therapy initiation. Chronic HCV infected persons were positive for HCV antibody (for >6 months) and HCV RNA and were seronegative for HIV (by enzyme-linked immunosorbent assay [ELISA]) and HBV (HBSag negative, HBVcore ab negative). HCV treated persons underwent DAA regimens (primarily Sofosbuvir/Ledipasvir, though also Sofosbuvir/Velpatasvir, Ombitasvir/Paritaprevir/Ritonavir, Dasabuvir/Ombitasvir/Paritaprevir/Ritonavir or Elbasvir/Grazoprevir) depending on the HCV genotype, drug/drug interactions, renal function, and prior treatment experience for a period of 8 or 12 weeks as per standard of care.

### Clinical Laboratory and Radiology Investigations

Certified clinical laboratories performed investigations to determine the extent of liver function; aspartate transaminase (AST), alanine transaminase (ALT) and albumin levels and liver damage index; Fibrosis 4 index (Fib-4) score (Age x AST level/Platelet count x √ALT level) and AST to platelet ratio index (APRI). Liver stiffness was determined by transient elastography (TE) in kilopaskals (kPa). Ten consecutive and successful measurements were performed per patient and only those obtained with a success rate of at least 60% and an interquartile range/median value (IQR/M) less than 30% were considered reliable. Cirrhosis status was determined here by participants having a TE score >12.5 kPa, anAPRI >1.5, or a liver biopsy consistent with cirrhosis.

### CD4+ T Cell Frequency and Absolute Count

PBMCs were labeled with anti- CD3-PERCP/CD4-Pacific Blue/CD8-APC-CY7/CD27-AF700/CD45RA-PE-CY7/CD31-BUV-395 (BD Biosciences, San Jose, CA). Absolute cell counts were obtained in fresh blood using Trucount™ absolute counting tubes (BD Biosciences, San Jose, CA). Flow cytometric analysis was performed on a BD LSRFortessa (BD Biosciences, San Jose, CA). Compensation was performed using single antibody labeled compensation beads; Live/Dead™ Fixable Aqua Dead cell stain-labeled Amine Reactive compensation beads (Life Technologies Corporation, Eugene, Oregon) and BD™ CompBeads (BD Biosciences, San Jose, CA) for cell-surface marker antibodies and analyzed with FACS DIVA software on the BD LSRFortessa.7 Population-based gating strategy was used to determine lymphocytes that were singlets and live cells, CD3+ T cells were divided into CD4+ and CD8+ subsets and thereafter CD4+ T cell subsets were defined by CD27 and CD45RA expression: naïve (CD27+CD45RA+), central memory (CM, CD27+CD45RA-) and effector memory (EM, CD27+CD45RA-) (**Supplemental Figure 1**). Naïve CD4+ T cell subsets were further defined as either CD31+ or CD31- based on isotype gating (21).

### Naïve CD4+ T Cell Isolation

Memory CD4+ T cells and non-CD4+ T cells were depleted from PBMCs by incubation with CD45RO, CD8, CD14, CD15, CD16, CD19, CD25, CD34, CD36, CD56, CD123, anti-TCRγ/δ, anti-HLA-DR, and CD235a (glycophorin A) antibodies (Miltenyi Biotech, Sunnyvale, CA). Naive CD4+ T cells were isolated using negative selection magnetic bead methods (Miltenyi Biotech, Sunnyvale, CA).

### Naive CD4+ T Cell Proliferation, Survival and Cell Death

Purified naïve CD4 T cells were cultured (10^5^ cells/200µL) in the presence and absence of 10 ng/ml of recombinant human IL-7 (Cytheris, Issy-les-Moulineaux, France) or 1 µl of ImmunoCult™ human CD3/CD28 T Cell Activator (Stemcell Technologies, Cambridge, MA) and incubated under conditions of 37°C and 5% CO_2_ for 5 days per condition. Cells were evaluated at days 0 (baseline), 3 and 5 of culture. Cells were washed and surface labeled in the dark at room temperature for 30 minutes with LIVE/DEAD™ Fixable Aqua Dead Cell Stain Kit (Thermo Fisher Scientific, Waltham, MA) and anti- CD3-PercP/CD4-Pacific Blue/CD8-APC-Cy7/CD27-AF700/CD45RA-PE-Cy7/CD31-BUV395/CD127-BV711 (BD Biosciences, San Jose, CA). Naive CD4+ T cells were identified based on expression of CD3, CD4, CD27 and CD45RA and lack of expression of CD8 markers and subsets were identified based on expression or absence of CD31. For detection of intracellular Ki67 (CD3/CD28 stimulation) and BCL-2 (IL-7 stimulation), cells were surface labeled, fixed, and permeabilized with a saponin-based buffer (BD Biosciences, San Jose, CA), followed by incubation with anti-Ki67-PE or anti-BCL-2- PE (BD Biosciences, San Jose, CA) for 40 minutes on ice. After labeling completion, cells were washed and fixed in phosphate-buffered saline containing 2% formaldehyde, and acquired on the BD LSRFortessa. For detection of apoptotic cells (after IL-7 stimulation), cells were labeled for 15 minutes with anti-AnnexinV-PE and anti-7AAD-FITC in Annexin V Binding Buffer (BD Biosciences, San Jose, CA) and evaluated on the BD LSRFortessa within 30 min of labeling completion.

### ELISA

Plasma from HCV infected and DAA-treated persons was assessed for inflammatory markers including soluble cluster of differentiation 14 (sCD14), interferon-inducible protein-10 (IP10), Autotaxin (ATX), sCD163, soluble Tumor Necrosis Factor Receptor II (sTNFRII) and IL-6; homeostatic cytokine IL-7; cytomegalovirus (CMV) IgG, by ELISA

(CMV ELISA kit from Diagnostic Automation/Cortez Diagnostics, Woodland Hills, CA and all other ELISA kits from R&D Systems, Minneapolis, MN).

### Statistical Analysis

Differences between two study groups were determined by Mann Whitney test and between two time-points within group by Related-Samples Wilcoxon Signed Rank tests. Associations between continuous variables per group were evaluated using Spearman’s rank correlation coefficient and Linear regression. Inter-group comparisons of linear regression lines were evaluated by covariance analysis. All tests were performed in GraphPad Prism, version 8 or SPSS for Windows v. 24.0 (IBM Corp, Armonk, New York). P value <0.05 considered statistically significant for all tests.

## Results

### Study Participant Characteristics

In the cross-sectional study, chronic HCV infected (n=34) and DAA-treated (n=29) persons and uninfected controls (n=25) were predominantly male and black (**Table 1**), consistent with our VA Northeast Ohio Healthcare system. HCV infected persons were age-distribution matched with controls (median 61 vs 59 years, p=0.6) and younger compared to DAA-treated persons (median 61 vs 65 years, p=0.01). The TE score (obtained prior to therapy) was higher in DAA-treated persons compared to active HCV infected persons (median 10.1 vs 5.0, p=0.0003). The HCV genotype 1A was predominant in both cross-sectional and longitudinal cohorts (**Table 1**). Additionally, features of the longitudinal cohort include an age similar to DAA-treated individuals in the cross-sectional cohort, and TE score similar to the untreated HCV cross-sectional group, and a modestly greater proportion of non-black males.

**Table 1:** Clinical characteristics of cross-sectional cohorts.

Sample size	HCV Infected n = 34	HCV Treated n = 29	Uninfected Control n = 25	P value Infected vs Treated	P value Infected vs Control	P value Treated vs Control
**Age, years**	61 (58; 65)	65 (62; 68)	59 (44; 71)	0.01*	0.7	0.2
**Gender; No. (%)**						
**Male**	32 (89%)	28 (97%)	22 (88%)			
**Female**	4 (11%)	1 (3%)	3 (12%)			
**Race; No. (%)**						
**Black**	21 (58%)	17 (60%)	18 (72%)			
**White**	13 (36%)	12 (40%)	6 (24%)			
**Other**	2 (6%)	0 (0%)	1 (4%)			
**Albumin level (g/dL)**	3.7 (3.5; 3.9)	3.8 (3.5; 4.0)	3.9 (3.7; 4.1)	0.5	0.04*	0.2
**ALT level (U/L)**	54 (35; 72)	25 (19; 31)	29 (25; 33)	<0.0001*	<0.0001*	NS
**AST level (U/L)**	43 (30; 63)	26 (18; 36)	21 (18; 24)	0.0004*	<0.0001*	NS
**Platelets (x10^9^/L)**	211 (162; 258)	190 (153; 232)	225 (201; 271)	0.3	0.2	0.02*
**APRI; No. (%)**						
**<0.4**	11 (37%)	10 (40%)	17 (89%)			
**0.4-1.5**	15 (50%)	14 (56%)	2 (11%)			
**>1.5**	4 (13%)	1 (4%)	0 (0%)			
**Fibrosis 4 index**	1.8 (1.3; 2.4)	1.9 (0.9; 3.2)	1.0 (0.8; 1.4)	0.9	0.002*	0.01*
**Transient Elastography score**	5.0 (4.4; 5.9)	10.1 (6.3; 21.9)	…	0.0003*	…	…
**Transient Elastography score (%)**						
**<9.5**	22 (92%)	7 (50%)	…			
**9.5-12.5**	1 (4%)	1 (7%)	…			
**>12.5**	1 (4%)	6 (43%)	…	,.		
**HCV Genotype; No. (%)**						
**1a**	24 (67%)	14 (47%)	…		…	…
**1b**	6 (17%)	8 (27%)	…		…	…
**2**	3 (8.5%)	4 (14%)	…		…	…
**3**	3 (8.5%)	1 (3%)	…		…	…
**4**	0 (0%)	1 (3%)	…		…	…
**Unknown**	0 (0%)	2 (6%)	…	…	…	…
**Plasma HCV RNA level (IU/L)**	4,816,448 (701,431; 4,355,261)	…	…	…	…	…

Median (25^th^, 75^th^ percentiles) shown unless otherwise indicated [No. (%)].

*Statistically significant (P value <0.05) using Mann Whitney test for unpaired comparison.

ALT, alanine aminotransferase; APRI, aspartate aminotransferase to platelet ratio index; AST, aspartate amino transferase; HCV, hepatitis C virus.

APRI calculated as AST/PLT.

Fibrosis 4 index calculated as [age × AST level]/[platelet count ×√ALT level].

### Naïve CD4+ Lymphopenia Is Associated With Cirrhosis and DAA HCV Therapy Is Associated With Partial Normalization

**Figure 1:**
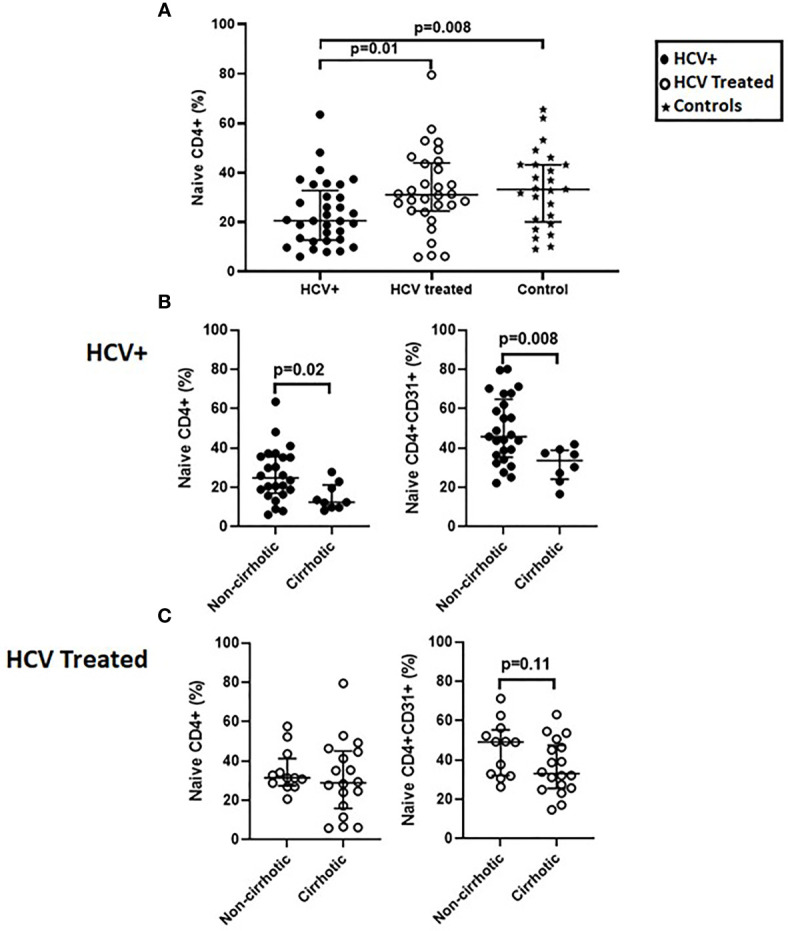
Naïve CD4+ proportions are lower in HCV infected persons compared to direct-acting antiviral (DAA) -treated persons and age-range matched uninfected controls and the HCV infected persons with cirrhosis have lower naïve CD4+ and CD4+CD31+ proportions compared to those without cirrhosis before and after DAA therapy. In a cross-sectional cohort study; chronic HCV infected persons (n=34), HCV DAA-treated persons at 1-5 years post-DAA therapy initiation (n=29) and age-range matched uninfected controls (n=25) we compared proportions (%) of lymphocyte gated T cells that were naïve CD4 T cells (CD3+CD4+ CD27+CD45RA+) **(A)**. The HCV infected and DAA-treated groups were stratified by cirrhosis status defined by Transient Elastography scores with cirrhotics: >12.5 kilopaskals (kPa) and non-cirrhotics: <12.5 kPa. Proportions of naïve CD4+ and naïve CD4+CD31+ T cells were assessed **(B, C)**. Mann Whitney p values shown when p<0.05.

We previously reported naïve CD4+ and CD4+CD31+ lymphopenia in chronic HCV infection (8). Here, we extended our initial observation, evaluating CD4+ T cell distribution in a cross-sectional cohort of chronic HCV infected (n=34) and DAA-treated (n=29) persons and age-range matched uninfected controls (n=25). In the cross-sectional analysis, naïve CD4+ proportions were lower in HCV infected persons compared to controls (p=0.008) and DAA-treated persons (p=0.01) (**Figure 1A**) while the CD4+CD31+ and CD4+CD31- proportions were comparable in all 3 groups (not shown). Naïve CD4+ (p=0.03), CD4+CD31+ (p=0.06) and CD4+CD31- (p=0.008) counts were lower in HCV infected persons compared to controls, though similar to DAA-treated persons (not shown). When stratified by cirrhosis status. The cirrhotics displayed lower naïve CD4+ (p=0.02) and CD4+CD31+ (p=0.008) proportions compared to non-cirrhotics in the HCV infected group (**Figure 1B**). In the DAA-treated group, cirrhotics tended to display lower naïve CD4+CD31+ (p=0.11) proportions compared to non-cirrhotics (**Figure 1C**).

In the longitudinal cohort analysis of chronic HCV infected persons before and after DAA therapy initiation (0, 4, 8 and 24 weeks), before therapy naïve CD4+ (p=0.04), CD4+CD31+ (p=0.02) and CD4+CD31+:CD4+CD31- ratio (p=0.03) proportions were lower in HCV infected persons compared to controls **Supplemental Figure 2A-C**), while CD4+CD31- proportions were higher (p=0.02) compared to controls (not shown). Within 24 weeks of DAA therapy initiation, there was a trend towards increased CD4+CD31+:CD4+CD31- ratios (p=0.18) (**Supplemental Figure 2C**) and trend for decreased CD4+CD31- frequency (p=0.18) (not shown). Further, before therapy absolute counts of naïve CD4+ (p=0.01), CD4+CD31+ (p=0.007) and CD4+CD31+:CD4+CD31- ratios (p=0.02) (**Supplemental Figures 2D–F**) and CD4+CD31- (p=0.03, not shown) were lower in HCV infected persons compared to controls. Within 24 weeks of DAA therapy initiation, there was a trend for increased CD4+CD31+:CD4+CD31- ratio (p=0.18) (**Supplemental Figure 2F**). Proportions of memory CD4+ T cell subsets remained unchanged after DAA initiation (not shown).

Collectively, these data indicate naïve CD4 lymphopenia is present during chronic active HCV infection, is localized to the CD31+ subset, and is more pronounced in those persons with cirrhosis. After therapy, naïve CD4+ and CD4+CD31+ lymphopenia appears partially normalized, though CD31+ subset frequency tends to remain lower in those with cirrhosis. The smaller longitudinal cohort data add further support for these findings.

### Age Is Associated With Naïve CD4+ T Cell Proportions in Untreated and Treated HCV Infection

**Figure 2:**
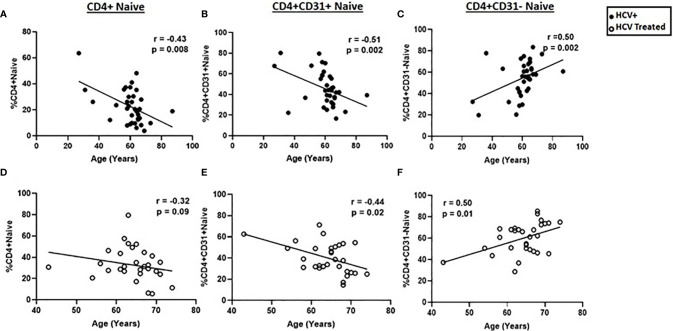
Age negatively correlates with naïve CD4+ and CD4+CD31+ proportions and positively correlates with naïve CD4+CD31- proportions. The correlations between age and the naïve CD4+, **(A, D)** CD4+CD31+ **(B, E)** and CD4+CD31- **(C, F)** T cell proportions were determined in chronic HCV infected (filled circles, n=34) and HCV DAA-treated (open circles, n=29) individuals. Spearman’s rank sum test was used; p= <0.05 considered significant.

Prior studies indicate that age is negatively associated with naïve CD4+CD31+ proportions in healthy individuals (11, 22, 23). We therefore evaluated the relation between age and naïve CD4 cells here, and determined if HCV treatment state impacted the relationship between age and naïve CD4+, CD4+CD31+ and CD4+CD31- counts and proportions in our cross-sectional cohort. In active HCV infection, age negatively correlated with naïve CD4+ (r=-0.43, p=0.008) and CD4+CD31+ (r=-0.51, p=0.002); proportions and positively correlated with CD4+CD31- proportions (r=0.50, p=0.002) (**Figure 2**). In the DAA-treated group this correlation between age and CD4+CD31+ (r=-0.44, p=0.02) and CD4+CD31- (r=0.5, p=0.006) subsets was preserved (**Figure 2**). Similarly, naïve CD4+ (r=-0.41, p=0.01) and CD4+CD31+ (r=-0.49, p=0.004) counts negatively correlated with age in active HCV infection. In DAA-treated persons, the correlation between naïve CD4+CD31+ counts and age (r=-0.40, p=0.03) was preserved. Naïve CD4+CD31- counts were not correlated with age in HCV infected or DAA-treated groups (not shown). Using linear regression, evaluating the interaction between age and naïve CD4+ cell subsets across groups, age associations with naïve CD4+ (p=0.96, -0.55 pooled slope), CD4+CD31+ (p=0.40, -0.74 pooled slope) and CD4+CD31- (p=0.40, 0.74 pooled slope) proportions did not differ between HCV infected and DAA-treated groups (**Supplemental Figure 3**), consistent with both age and HCV infection status driving naïve CD4+ cell lymphopenia. Naïve CD4+ T cell frequency/count and subset distribution did not differ by HCV genotype, sex or race.

### Low Albumin Level, Liver Inflammation, Fibrosis, HCV Level and Soluble CD14 Level Are Associated With Naïve CD4+ and CD4+CD31+ Lymphopenia

Liver disease, regardless of etiology, may result in naïve T cell loss (8, 22, 24). Although splenic sequestration is one possible underlying mechanism (25), other factors may contribute. Chronic HCV infection contributes to liver inflammation and fibrosis, and impaired liver function, which in turn are associated with elevated systemic soluble immune activation markers (26). To better understand mechanisms underlying naïve CD4+ lymphopenia, we evaluated relationships between naïve CD4+ T cell numbers and plasma HCV level, markers of ongoing liver damage (ALT, AST), liver stiffness/fibrosis (TE, APRI, and FIB-4), liver synthetic function (albumin) and markers of systemic immune activation (sCD14, sCD163, ATX, TNFRII, IL-6 and IP-10 plasma levels). IP-10 levels (p=0.002) were elevated in HCV infected persons compared to DAA-treated persons while the levels of sCD14, sCD163, ATX, TNFRII and IL-6 were comparable between the two groups (**Supplemental Table 2**). FIB-4 score tended to negatively correlate with naïve CD4+ proportions (r=-0.35, p=0.06) and negatively correlated with CD4+CD31+ counts (r=-0.38, p=0.04), while HCV level tended to negatively correlate with naïve CD4+ counts (r=-0.30, p=0.06) in HCV infected persons. TE score negatively correlated with CD4+CD31+ proportions (r=-0.63, p=0.02) in DAA-treated persons. Albumin level positively correlated with CD4+CD31+ counts (r=0.60, p=0.01) in controls and CD4+CD31+ proportions (r=-0.63, p=0.02) in DAA-treated persons. No other correlations were observed in controls. For immune activation markers, sCD14 negatively correlated with naïve CD4+ proportions (r=-0.45, p=0.02) in DAA-treated persons. No correlations were observed for the CD4+CD31- subset. Collectively, naïve CD4+ and CD4+CD31+ lymphopenia was associated with liver inflammation and fibrosis (FIB-4 and TE scores), immune activation (sCD14 level) and low liver albumin synthesis in chronic HCV infection even after DAA therapy.

### *Ex vivo* Apoptosis in Naïve CD4+CD31+ T Cells During Chronic HCV Infection Is Greater Compared to DAA-Treated HCV

To address HCV infection mediated mechanisms of naïve CD4+ T cell homeostasis, we examined two important functions; naïve CD4+ T cell direct *ex vivo* cell death and their death and capacity to respond to media vs. interleukin-7 (IL-7) *in vitro*. IL-7 is a homeostatic cytokine that can enhance the peripheral expansion and survival of naïve CD4+ T cells (27, 28), or TCR stimulus. We determined whether DAA therapy for chronic HCV infection was associated with naïve CD4+ T cell survival by direct *ex vivo* AnnexinV and 7AAD labeling, expression of pro-survival factor BCL-2 and IL-7R (CD127), or response to *in vitro* IL-7 stimulation. Furthermore, TCR induced activation of naïve CD4+ T cells is the first of three T cell activation signals (in addition to co-stimulation and cytokine-mediated differentiation) and impaired T cell activation following antigenic-stimulation during chronic HCV infection may dampen vaccine responses. We therefore evaluated the capacity of each naïve CD4+ T cell subset to undergo cell cycling (Ki67 labeling) following TCR stimulus before and after treatment of chronic HCV infection.

**Figure 3:**
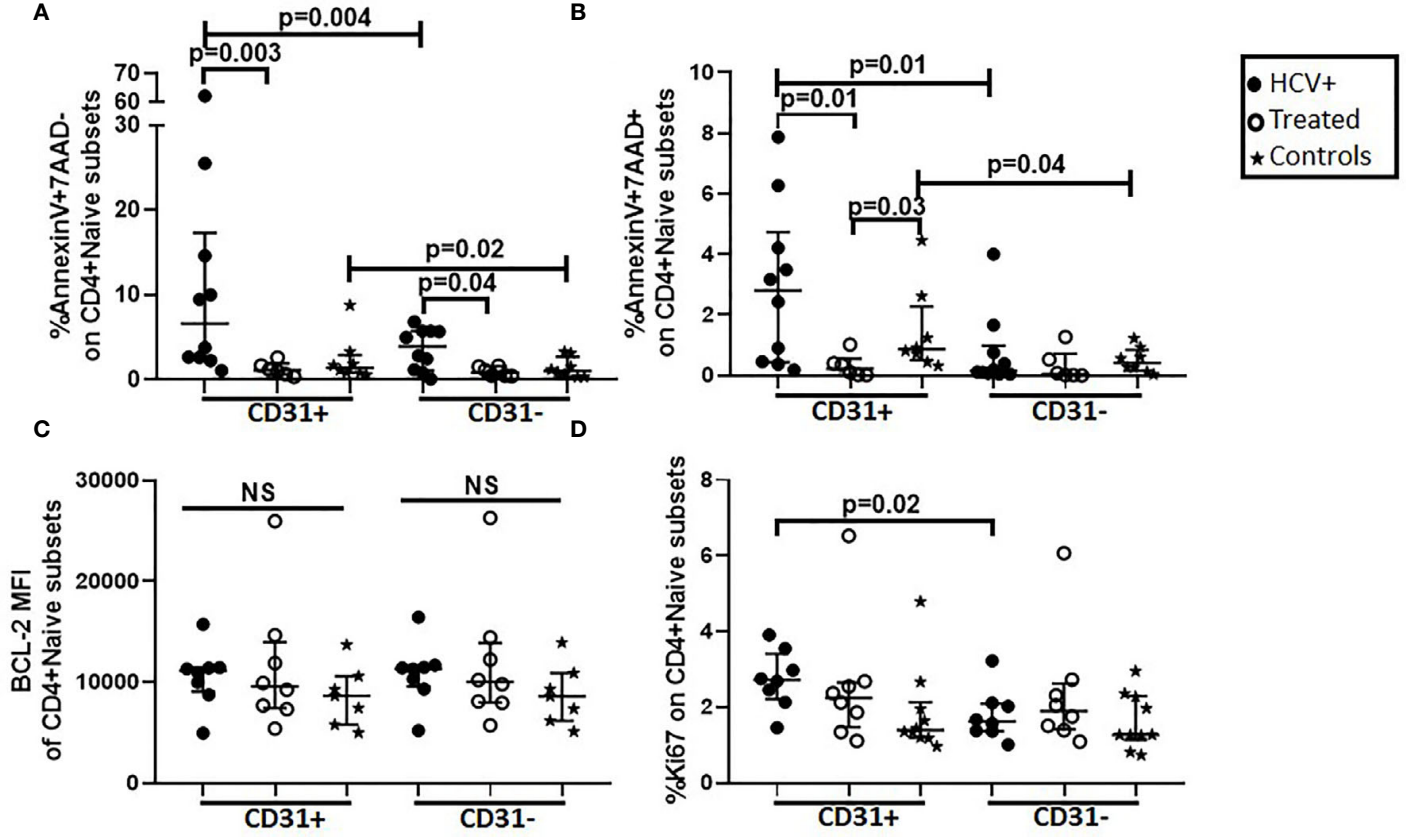
Direct *ex vivo* apoptosis in CD31+ and CD31- naïve CD4+ T cell subsets is greater in HCV infected individuals compared to HCV DAA-treated individuals and uninfected controls and the CD4+CD31+ subset exhibits greater *ex vivo* apoptosis and cell cycling (Ki67 expression) compared to the CD4+CD31- subset. Magnetic bead purified (negative selection) naïve CD4 T cells from chronic HCV infected (filled circles, n=8), HCV DAA-treated (open circles, n=8) and age-range matched uninfected control (stars, n=7) groups were analyzed by flow cytometry for apoptosis (AnnexinV and 7AAD) **(A, B)**, BCL-2 **(C)** and cell cycling (Ki67) **(D)** on naïve (CD27+CD45RA+) CD4+CD31+ and CD4+CD31- T cells. Mann Whitney test was used for comparisons between two groups and between the CD4+CD31+ and CD4+CD31- subsets; p= <0.05 considered significant. NS represents non-significant p values.

The early and late stages of cell death can be detected using AnnexinV (binds phosphatidylserine on outer leaflet of plasma membrane of apoptotic cells) and 7AAD (penetrates dead and apoptotic cells to bind double-stranded nucleic acids) respectively. Using AnnexinV and 7AAD, we evaluated the susceptibility of naïve CD4+ subsets to undergo cell death *in vivo* by examining purified naïve CD4+ cells and PBMCs for direct *ex vivo* apoptosis in naïve (CD31+ and CD31-) and memory (CM and EM) CD4+ T cell subsets respectively in HCV-infected (n=8) and DAA-treated (n=6) persons and controls (n=8). Cells from HCV infected persons exhibited greater AnnexinV+7AAD- labeling (early-stage apoptosis) in naïve (CD31+ p=0.03, CD31- p=0.04; **Figure 3A**) and memory (CM p=0.03, EM p=0.005; not shown) T cell subsets compared to DAA-treated persons and controls respectively. Compared to CD4+CD31-, CD4+CD31+ T cells displayed greater AnnexinV+7AAD- and AnnexinV+7AAD+ (late-stage apoptosis) labeling in HCV infected persons (p=0.004, p=0.01) and controls (p=0.02, p=0.04) (**Figures 3A, B**). These data are consistent with greater AnnexinV+7AAD+ CD4+CD31+:CD4+CD31- T cell ratios in HCV infected persons compared to DAA-treated persons (p=0.04, not shown). We also investigated the spontaneous apoptosis of naïve CD4+ subsets by examining purified CD4+ naïve T cells with or without IL-7 stimulation after 3 days. The IL-7 stimulated CD4+CD31- T cells displayed lower AnnexinV+7AAD+ labeling compared to media treated CD4+CD31- T cells from the HCV infected (p=0.02), DAA-treated (p=0.03) and control (p=0.02) persons while no differences were observed for the CD4+CD31+ T cells (**Supplemental Figure 4**). Further, media treated CD4+CD31- T cells displayed greater levels of AnnexinV+7AAD- labeling compared to media treated CD4+CD31+ T cells from the HCV infected (p=0.03) and DAA-treated persons (p=0.03) and controls (p=0.008) (not shown).

To further delineate mechanisms of apoptosis, we examined intracellular levels of pro-survival factor; BCL-2, and Ki67 (cell cycling) in purified naïve CD4+ cells *ex vivo*. Naïve CD4+ subsets were all BCL-2 positive and the median fluorescence intensity (MFI) enabled the differentiation of the magnitude of BCL-2 expression MFI was comparable in gated naïve CD4+ T cell subsets across the 3 study groups (**Figure 3C**). Ki67 labeling was greater in CD4+CD31+ compared to CD4+CD31- naïve T cells (p=0.02) in HCV infected persons (**Figure 3D**). Inter-group comparisons revealed HCV infected persons exhibited greater in CD4+CD31+:CD4+CD31- Ki67+ T cell ratios when compared to DAA-treated persons (p=0.03, not shown). Together, the data demonstrated more enhanced *ex vivo* early-stage apoptosis in naïve and memory CD4+ T cells in HCV infection compared to treated HCV and controls respectively, and greater *ex vivo* late-stage apoptosis and cell cycling in CD4+CD31+ compared to CD4+CD31- subsets in HCV infection and controls.

### IL-7 Induced BCL-2 Expression in Naïve CD4+ T Cell Subsets Is Greater in Chronic HCV Infected Persons Compared to DAA-Treated Persons

**Figure 4:**
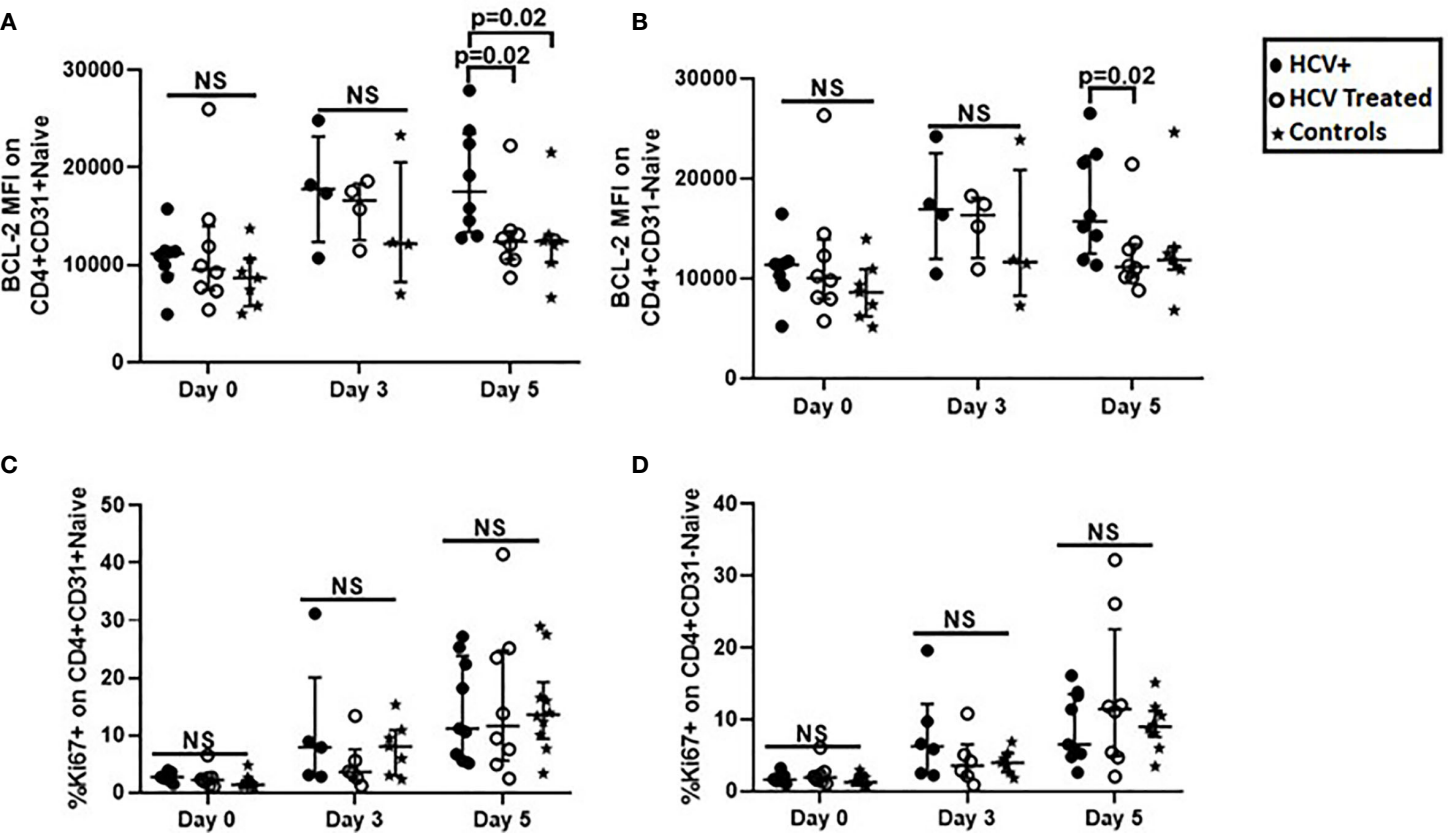
BCL-2 expression after IL-7-stimulation was greater in CD31+ and CD31- naïve CD4+ T cell subsets from HCV infected persons compared to naïve CD4+ T cells from HCV DAA-treated persons and uninfected controls, while T cell Receptor-dependent cell cycle entry was similar in naïve CD4+ T cell subsets in all three groups. Magnetic bead purified (negative selection) naïve CD4 T cells from chronic HCV infected (filled circles, n=8), HCV DAA-treated (open circles, n=8) and age-range matched uninfected control (stars, n=7) groups were stimulated with 10ng/ml of recombinant human IL-7 or 1ul anti-CD3/anti-CD28 Activator for 5 days. On 0, 3, and 5 days, flow cytometric analysis of BCL-2 following IL-7 stimulation **(A, B)** and Ki67 following anti-CD3/anti-CD28 stimulation **(C, D)** on naïve (CD27+CD45RA+) CD4+CD31+ and CD4+CD31- T cells was performed. Mann Whitney test was used for comparisons between two groups; p= <0.05 considered significant. NS represents non-significant p values.

We next investigated if *in vitro* 5-day stimulation with recombinant IL-7 or CD3/CD28 activator would impact survival (apoptosis and BCL-2 expression), and cell cycling (Ki67 labeling) in our cross-sectional cohort. At Day 5 of IL-7 stimulation, BCL-2 upregulation in CD4+CD31+ cells of HCV infected persons was greater compared to cells of HCV treated persons (p=0.02) and controls (p=0.02, **Figure 4A**). BCL-2 upregulation in CD4+CD31- cells of HCV infected persons was also greater compared to cells of HCV treated persons (p=0.02), but similar to controls (**Figure 4B**). To determine if IL-7R (CD127) expression levels contributed to differential IL-7-induced BCL-2 upregulation between the groups, we examined IL-7R (CD127) levels on naïve CD4+ subsets. At baseline (Day 0), IL-7R MFI or positive% of naïve CD4+ subsets were comparable between the 3 study groups. In addition, the CD4+CD31+ subsets displayed greater IL-7R MFI compared to CD4+CD31- subsets in HCV infection (p=0.008) and treated HCV (p=0.03) (not shown). Following 5-day IL-7 stimulus, no inter-group or naïve subset differences in IL-7R down-regulation were observed (not shown). Plasma IL-7 levels were also comparable across groups (not shown).

At baseline (Day 0), no inter-group differences in bulk naïve CD4+ subset Ki67 labeling were observed (**Figure 4B**), however, Ki67+ CD4+CD31+:CD4+CD31- T cell ratios were greater in HCV infection compared to treated HCV (p=0.03, not shown). Following 5-day TCR stimulus (CD3/CD28), no inter-group differences in naïve CD4+ subset Ki67 labeling were observed (**Figures 4C, D**). Notably, CD31 expression on naïve CD4+ T cells was similar after stimulation with IL-7 or CD3/CD28 activator, with exception of a modest increase in CD31 after 5 days of CD3/CD28 stimulation for control subject samples (**Supplementary Figure** for reviewer only.

Overall, IL-7-induced BCL-2 up-regulation in naïve CD4+ subsets was greater in HCV infected persons compared to HCV-treated and control persons, while IL-7R down-regulation was similar across groups. At baseline, cell cycling in CD31+ relative to CD31- subsets was greater in active HCV infection, and no differences in cell cycling were observed between groups before or after TCR stimulus.

## Discussion

In the present study, we observed lower naïve CD4+ T cell proportions in chronic HCV infected persons compared to DAA-treated persons and age-range matched uninfected controls. Further, HCV infected persons with cirrhosis displayed lower naïve CD4+ and CD4+CD31+ T cell proportions compared to those without cirrhosis before DAA therapy and this partially normalized when evaluating persons treated with DAA therapy. Results were consistent in the much smaller longitudinal cohort. Age negatively correlated with naïve CD4+ and CD4+CD31+ proportions, and positively correlated with CD4+CD31- proportions before and after HCV DAA therapy. Direct *ex vivo* apoptosis in naïve and memory CD4+ T cells was elevated in chronic HCV infection, and the CD4+CD31+ subset exhibited greater apoptosis and cell cycling compared to the CD4+CD31- naïve subset. *In vitro* IL-7-induced BCL-2 upregulation was greater in naïve CD4+ cells from HCV infected persons compared to cells from HCV-treated persons and controls. Taken together, chronic HCV infection state, cirrhosis state, and age impact naïve CD4+ T cell proportions, likely by differing mechanisms. DAA therapy is associated with numerical and fractional normalization of naïve CD4+ lymphopenia. Plausible mechanisms underlying naïve CD4+ lymphopenia attributable to HCV infection include enhanced cellular apoptosis and activation (cycling), more so in the recent thymic emigrant CD4+CD31+ subset.

Naïve CD4+ and CD4+CD31+ T cell lymphopenia in chronic HCV infection was previously reported by our group and others (8, 12, 29). Here, we confirm this prior literature and extend previous reports to the impact of DAA treatment on naïve CD4+ T cell lymphopenia. Before therapy the proportions and counts of naïve CD4+, CD4+CD31+ and CD4+CD31- T cells in chronic HCV infected persons were lower compared to controls. DAA therapy was associated with partial normalization of naïve CD4+ proportions to levels observed in age-range matched controls, with a residual trend toward lower naïve CD4+CD31+ frequencies in cirrhotics compared to non-cirrhotics (**Figure 1**).

Age is also associated with naïve CD4+ T cell lymphopenia, largely thought to be due to thymic involution and decreased thymopoiesis in older individuals with subsequent reduction of naïve CD4+CD31+ T cell export into the periphery (7, 11). Here, associations between age and both proportions and counts of naïve CD4+ T cells and corresponding subsets were present both before and after DAA therapy, indicating that the age-related mechanisms regulating naïve CD4+ T cell homeostasis are observed regardless of, and perhaps independent of, HCV treatment status, suggesting different mechanisms. Indeed, data provided here indicate HCV mediated cell death of the naïve CD4+ T cell pool, contrasting with reduced thymic output in aged persons. Notably, age was negatively correlated with CD4+CD31+ counts but not correlated with CD4+CD31- counts regardless of HCV infection state, consistent with an age effect selective for the CD31+ subset. Overall, mechanisms of both age- and HCV- driven (reversed with DAA) lymphopenia were operative in our cohorts of chronic HCV infected persons of older age.

Mechanisms underlying HCV-associated naïve CD4+ lymphopenia have not been clearly defined. One potential mechanism is naïve CD4+ T cell anatomic redistribution due to portal hypertension and splenic sequestration (25), however, this does explain the selective association of HCV infection with lymphopenia in the CD4+CD31+ but not CD4+CD31- subset. Alternatively, insufficient DNA (deoxyribonucleic acid) repair enzyme can result in naïve CD4+ T cell accumulation of damaged DNA triggering intrinsic apoptosis and T cell loss during HCV infection (30). Here, since 7AAD binds to intra-cytoplasmic double-stranded nucleic acid fragments in apoptotic cells, the observed enhanced Annexin+7AAD+ labeling of naïve CD4+ T cells (direct *ex vivo*) from HCV infected persons could be consistent with this mechanism. At the same time direct *ex vivo* analysis of BCL-2 expression within naïve CD4+ T cells was similar between untreated and treated HCV and control persons, consistent with prior studies in chronic HCV (31–33) and suggesting that alteration of this intrinsic anti-apoptotic mechanism is not likely operative here. In fact *in vitro* media cultured cell apoptosis (measure of intrinsic resistance to apoptosis) does not appear to remarkably differ between groups but as expected resistance is enhanced by IL-7 culture (34, 35). Notably after IL-7 culture, HCV infected person cells exhibited greater BCL-2 expression compared to HCV-treated person cells, while IL-7-induced IL-7R downregulation was similar in cells from the two groups.

Another potential mechanism of HCV infection associated naïve CD4+ lymphopenia is activation-induced cell death, particularly in the CD4+CD31+ subset, following antigen specific or non-specific activation. In support of this, we observed greater late-stage (AnnexinV+7AAD+) apoptosis and cycling (Ki67 expression) in CD4+CD31+ compared to CD4+CD31- T cells in HCV infected persons and controls. Non-antigen specific naïve CD4+ T cell activation in HCV infection may be mediated by HCV envelope protein E2 interaction with cell surface receptor CD81 on naïve T cells (36) or by soluble immune activation factors secreted by inflamed livers. In support, we observed a negative correlation between sCD14 level (released from activated monocytes and Kupffer cells) and naïve CD4+ proportions in DAA-treated persons. Notably, sCD14 plasma levels were similar between HCV untreated and treated persons, consistent with previous reports (26, 37), raising the possibility that residual liver inflammation after DAA therapy is one possible driver of naïve CD4 lymphopenia, while other factors are likely also involved before DAA therapy. Notably, lower liver biosynthetic function (albumin) and elevated liver inflammation and fibrosis (Fib-4 and TE scores) levels were associated with lower naïve CD4+ and CD4+CD31+ numbers before and after DAA therapy, demonstrating that the long-term effects of HCV infection and advanced liver damage may persist despite HCV cure possibly contributing to mechanisms that interfere with naïve CD4+ T cell homeostasis. Here, cirrhotics exhibited more naïve CD4+ and CD4+CD31+ lymphopenia during HCV infection, a finding that persisted despite effective DAA treatment, consistent with previous reports of CD4+CD31+ lymphopenia during liver cirrhosis regardless of cause (22, 38).

Lastly, the extrinsic apoptotic pathway *via* surface death receptors (including Fas receptor) may also contribute to naïve CD4+ T cell lymphopenia. HCV infection has been described to associate with greater Fas expression on peripheral bulk T cells, greater serum FasL levels, and apoptosis of bulk peripheral T cells (32, 39, 40). The liver may be one source of peripheral FasL since it is highly expressed within the liver immune cells in HCV infection (41). While these studies did not specifically evaluate naïve CD4+ T cells, it is likely this mechanism contributes here. Indeed, literature from the mouse system suggests naïve T cells may be more susceptible to Fas-mediated apoptosis following antigenic stimulation (33), and this is consistent with our observation of late-stage (Annexin+ 7AAD+) apoptosis in only naïve but not memory CD4+ T cells.

The current study has a number of limitations. The study participants are reflective of the North East Ohio VA population with African-American and male predominance. Female and non-black populations were under-represented and further study is needed to understand how these results extend to the general U.S. population. Investigations of longitudinal changes after DAA therapy initiation were severely limited by small sample size and inconsistent follow-up. Relationships between age and naïve CD4+ T cell numbers in the uninfected controls did not reach significance here, contrasting with published data, also perhaps due to small sample size or additional confounding factors in our sample set. Our measurements were peripheral blood based, and perhaps limited in ability to reflect events within the liver and thymus. However, these results build upon prior literature, focusing here on naïve CD4+ subsets, effects of DAA therapy on naïve CD4+ homeostasis, and mechanisms of cellular activation and cell death associated with DAA treatment status.

In summary, we have described naïve CD4+ T cell lymphopenia and apoptosis, associated with cell cycling in the CD31+ naïve CD4 T cell compartment, that is partially normalized after initiation of DAA therapy in chronic HCV infection. Age related naïve CD4 lymphopenia appears to be the result of alternative mechanism, such as reduced thymic output, and this is superimposed upon the state of chronic HCV infection and apoptosis. The downstream effect of restoration of this compartment after HCV DAA therapy is yet to be determined.

## Data Availability Statement

The original contributions presented in the study are included in the article/**Supplementary Material**. Further inquiries can be directed to the corresponding author.

## Ethics Statement

The studies involving human participants were reviewed and approved by VA Northeast Ohio Healthcare System Institutional Review Board (IRB). The patients/participants provided their written informed consent to participate in this study.

## Author Contributions

Study concept and design: DA, AA CS, DC, and PP. Technical support and Manuscript review: AL, SD, CK, and EZ. Data acquisition: AA and AL. Data analysis and interpretation: AA and DA. Statistical analysis: AA, BW, and DA. Manuscript drafting: AA and DA. Critical revision of manuscript: AA, DA, CS, DC, and PP. Study supervision: AA, DA and CS. All authors contributed to the article and approved the submitted version.

## Funding

This study was Supported by D43TW010319 (AA), IK2CX001471 (CS), BX001894 (DA), CX001791 (DA).

## Conflict of Interest

The authors declare that the research was conducted in the absence of any commercial or financial relationships that could be construed as a potential conflict of interest.

## Supplementary Material

Supplementary Table 1 Clinical characteristics of HCV DAA-treated longitudinal cohort. Median (25^th^;75^th^ percentiles) shown unless otherwise indicated.

Supplementary Table 2 Soluble markers of systemic immune activation markers of cross-sectional cohorts. Median (25^th^; 75^th^ percentiles). *Statistically significant (P value <0.05) using Mann Whitney test for unpaired comparison. **Samples not available for full cohort

Supplementary Figure 1 Flow cytometry gating strategy for naïve CD4+ T cells and corresponding subsets.

Supplementary Figure 2 Naïve CD4+, CD4+CD31+ and CD4+CD31- proportions and counts are lower in HCV infected individuals compared to age-range matched uninfected controls and initiation of direct-acting antiviral (DAA) therapy tended to increase naïve CD4+ and CD4+CD31+ but not CD4+CD31- counts. In a longitudinal cohort study, chronic HCV infected individuals were treated with 8 or 12 weeks of DAA therapy and followed from baseline (start; n=16) to time-points after DAA therapy initiation; Weeks 4 (n=13), 8 (n=6) and 24 (n=6). Proportions **(A–C)** and counts **(D–F)** of naïve CD4+ T cells (A and D), CD4+CD31+ T cells **(B, E)** and CD431+:CD31- T cell ratios **(C, F)** were assessed. Age-range matched uninfected controls (n=25) we compared for each T cell parameter. Wilcoxon signed rank test was used for paired comparisons between two time points; p= <0.05 considered significant.

Supplementary Figure 3 The degree of association between age and the naïve CD4+, CD4+CD31+ and CD4+CD31- proportions does not significantly differ before and after HCV DAA therapy. The associations between age and the naïve CD4+, CD4+CD31+ and CD4+CD31- T cell proportions in the chronic HCV infected (filled circles, n=34) and HCV DAA-treated (open circles, n=29) groups and the differences between the two study groups were determined. R and p values for correlations within each group (HCV infected and HCV treated) are shown, and p values to determine differences in correlations between groups are shown. We can discuss whether we want p value for y axis intercept as well. The Linear regression test was used.

Supplementary Figure 4 The naïve CD4+CD31- T cells undergo spontaneous apoptosis at higher levels compared to the naïve CD4+CD31+ T cells before and after HCV DAA therapy and in absence of HCV infection. Magnetic bead purified (negative selection) naïve CD4 T cells from chronic HCV infected (filled circles, n=4), HCV DAA-treated (open circles, n=4) and age-range matched uninfected control (stars, n=5) groups were stimulated with or without 10ng/ml of recombinant human IL-7 for 3 days. On third day, flow cytometric analysis for apoptosis (AnnexinV and 7AAD) on naïve (CD27+CD45RA+) CD4+CD31+ **(A)** and CD4+CD31- **(B)** T cells was performed. Mann Whitney test was used for comparisons between two groups; p= <0.05 considered significant.

Supplementary Figure 5 CD31 expression on naïve CD4+ T cells is mostly stable during *in vitro* stimulation with recombinant IL-7 or CD3/CD28 activator in cells from HCV infected and DAA-treated persons. Magnetic bead purified (negative selection) naïve CD4 T cells from chronic HCV infected (filled circles, n=8; **A, D**), HCV DAA-treated (open circles, n=8; **B, E**) and age-range matched uninfected control (stars, n=7; **C, F**) groups were stimulated with 10ng/ml of recombinant human IL-7 **(A–C)** or 1ul anti-CD3/anti-CD28 Activator **(D–F)** for 5 days. On 0 (direct *ex vivo*), 3, and 5 days, flow cytometric analysis of CD31 expression on naïve (CD27+CD45RA+) CD4+ T cells was performed. Mann Whitney test was used for comparisons between two groups; p= <0.05 considered significant.
