# Supplementary material for: Naïve CD4+ T Cell Lymphopenia and Apoptosis in Chronic Hepatitis C Virus Infection Is Driven by the CD31+ Subset and Is Partially Normalized in Direct-Acting Antiviral Treated Persons
Source: Front Immunol. 2021 Apr 12;12:641230. doi: 10.3389/fimmu.2021.641230 (PMC8075159; doi:10.3389/fimmu.2021.641230)
Supplement: Supplementary Table 1 — Clinical characteristics of HCV DAA-treated longitudinal cohort. Median (25th;75th percentiles) shown unless otherwise indicated. [file Table_1.pdf]

Supplemental Table 1: **Clinical characteristics of HCV DAA-treated longitudinal cohort**

**Parameters**	**HCV Infected at DAA Therapy Start n=16**
**Age, years[1]**	64 (58-67)
**Gender; No. (%)**	
**Male**	15 (94%)
**Female**	1 (6%)
**Race/ethnicity; No. (%)**	
**Black**	9 (44%)
**White**	7 (56%)
**Other**	0 (0%)
**Albumin level (g/dL)**	3.6 (3.5;3.9)
**ALT level (U/L)**	45 (35; 61)
**AST level (U/L)**	31 (30; 45)
**Platelets ($x10^9$/L)**	225 (186; 279)
**APRI; No. (%)**	
**<0.4**	6 (55%)
**0.4-1.5**	4 (36%)
**>1.5**	1 (9%)
**Fibrosis 4 index**	1.7 (1.3; 2.4)
**Transient Elastography score**	5 (4.4,6.0)
**Transient Elastography score (%)**	
**<9.5**	11 (100%)
**9.5-12.5**	0 (0%)
**>12.5**	0 (0%)
**HCV Genotype; No. (%)**	
**1a**	11 (69%)
**1b**	3 (19%)

**2**	2 (12%)
**Plasma HCV RNA level (IU/L)**	3,014,233 (311,195; 4,767,721)

Median (25th;75th percentiles) shown unless otherwise indicated
