# Supplementary material for: Naïve CD4+ T Cell Lymphopenia and Apoptosis in Chronic Hepatitis C Virus Infection Is Driven by the CD31+ Subset and Is Partially Normalized in Direct-Acting Antiviral Treated Persons
Source: Front Immunol. 2021 Apr 12;12:641230. doi: 10.3389/fimmu.2021.641230 (PMC8075159; doi:10.3389/fimmu.2021.641230)
Supplement: Supplementary Table 2 — Soluble markers of systemic immune activation markers of cross-sectional cohorts. Median (25th; 75th percentiles). *Statistically significant (P value <0.05) using Mann Whitney test for unpaired comparison. **Samples not available for full cohort [file Table_2.pdf]

Supplemental Table 2: Soluble markers of systemic immune activation markers of cross-sectional cohorts

Sample size**	HCV Infected n=exact sample size	HCV Treated n=	P value
**IP-10 (pg/mL)**	56.2 (36.8; 104.1)	25.8 (17.4; 34.5)	0.002*
**Soluble CD163 (ng/mL)**	26.7(18.2; 40.0)	19.0 (14.9; 25.5)	0.09
**Autotaxin (ng/mL)**	15.0 (0.02; 23.0)	17.4 (13.8; 22.6)	0.50
**Soluble CD14 (ng/mL)**	4996 (4314; 7242)	4999 (3662; 6102)	0.76
**Soluble TNFRII (pg/mL)**	70.0 (51.5; 80.2)	75.8 (49.9; 96.0)	0.83
**Il-7 (pg/mL)**	1.0 (10.0; 23.0)	1.2 (0.8; 1.9)	0.48
**IL-6 (pg/mL)**	3.4 (0.5;1.6)	4.2 (3.4; 5.6)	0.27

Median (25th; 75th percentiles)

*Statistically significant (P value <0.05) using Mann Whitney test for unpaired comparison

**Samples not available for full cohort
