# Supplementary figures and images for: Naïve CD4+ T Cell Lymphopenia and Apoptosis in Chronic Hepatitis C Virus Infection Is Driven by the CD31+ Subset and Is Partially Normalized in Direct-Acting Antiviral Treated Persons
Source: Front Immunol. 2021 Apr 12;12:641230. doi: 10.3389/fimmu.2021.641230 (PMC8075159; doi:10.3389/fimmu.2021.641230)

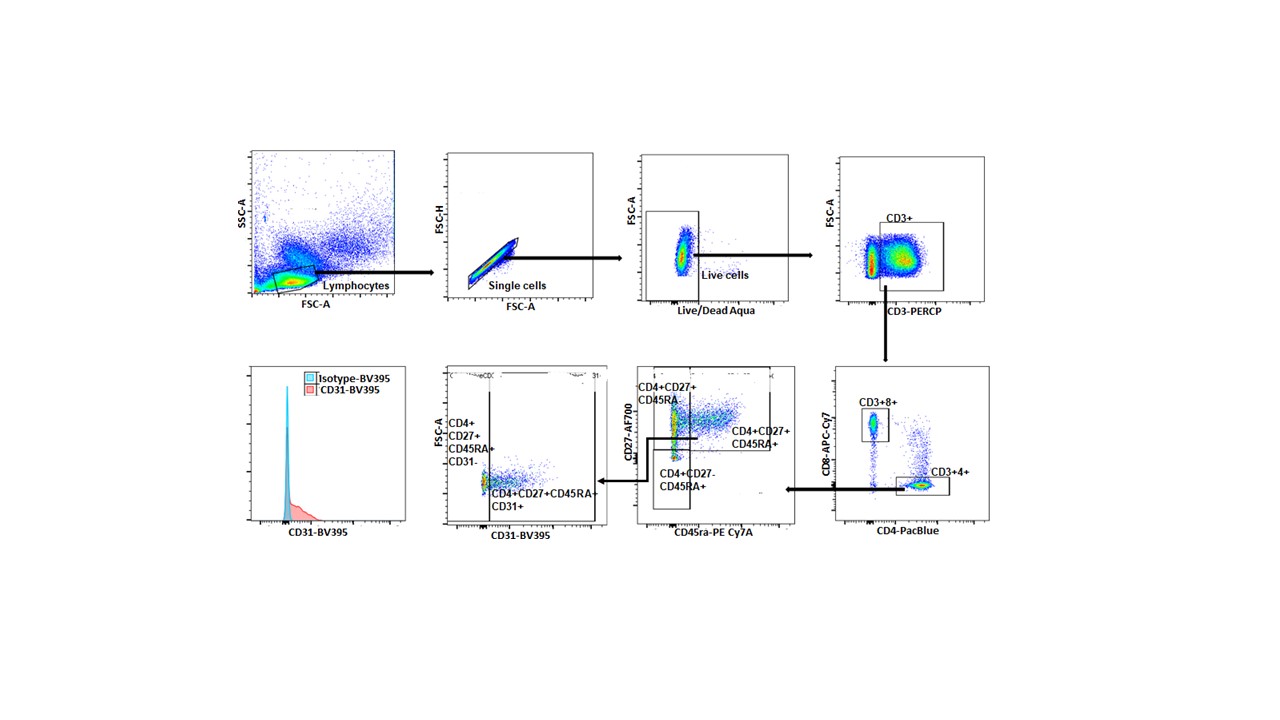

Supplement: Supplementary Figure 1 — Flow cytometry gating strategy for naïve CD4+ T cells and corresponding subsets. [file Image_1.jpeg]

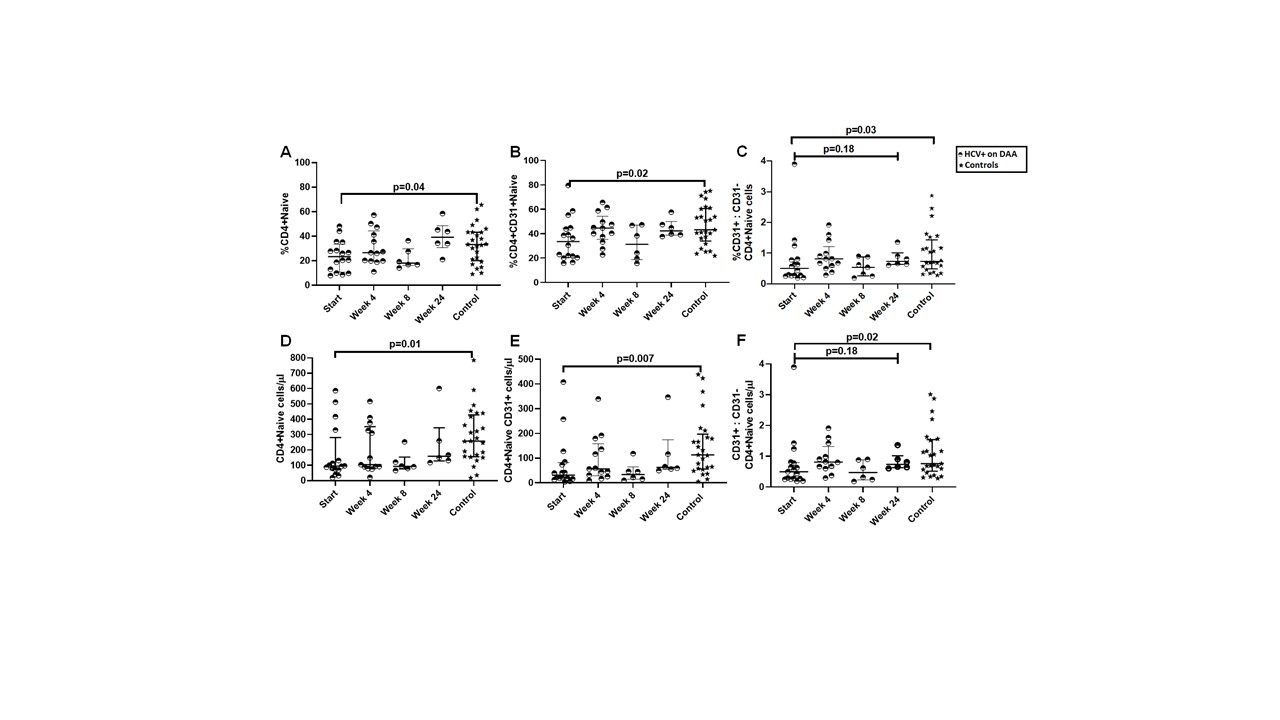

Supplement: Supplementary Figure 2 — Naïve CD4+, CD4+CD31+ and CD4+CD31- proportions and counts are lower in HCV infected individuals compared to age-range matched uninfected controls and initiation of direct-acting antiviral (DAA) therapy tended to increase naïve CD4+ and CD4+CD31+ but not CD4+CD31- counts. In a longitudinal cohort study, chronic HCV infected individuals were treated with 8 or 12 weeks of DAA therapy and followed from baseline (start; n=16) to time-points after DAA therapy initiation; Weeks 4 (n=13), 8 (n=6) and 24 (n=6). Proportions (A–C) and counts (D–F) of naïve CD4+ T cells (A and D), CD4+CD31+ T cells (B, E) and CD431+:CD31- T cell ratios (C, F) were assessed. Age-range matched uninfected controls (n=25) we compared for each T cell parameter. Wilcoxon signed rank test was used for paired comparisons between two time points; p= <0.05 considered significant. [file Image_2.jpeg]

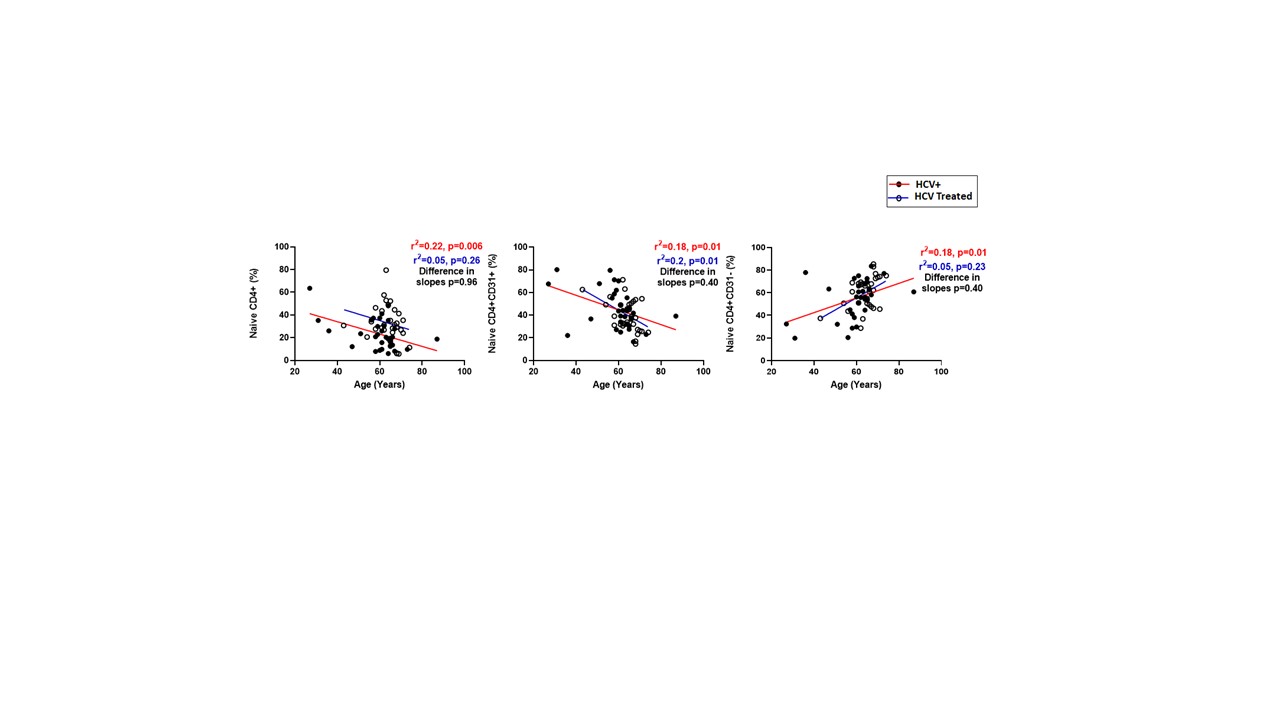

Supplement: Supplementary Figure 3 — The degree of association between age and the naïve CD4+, CD4+CD31+ and CD4+CD31- proportions does not significantly differ before and after HCV DAA therapy. The associations between age and the naïve CD4+, CD4+CD31+ and CD4+CD31- T cell proportions in the chronic HCV infected (filled circles, n=34) and HCV DAA-treated (open circles, n=29) groups and the differences between the two study groups were determined. R and p values for correlations within each group (HCV infected and HCV treated) are shown, and p values to determine differences in correlations between groups are shown. We can discuss whether we want p value for y axis intercept as well. The Linear regression test was used. [file Image_3.jpeg]

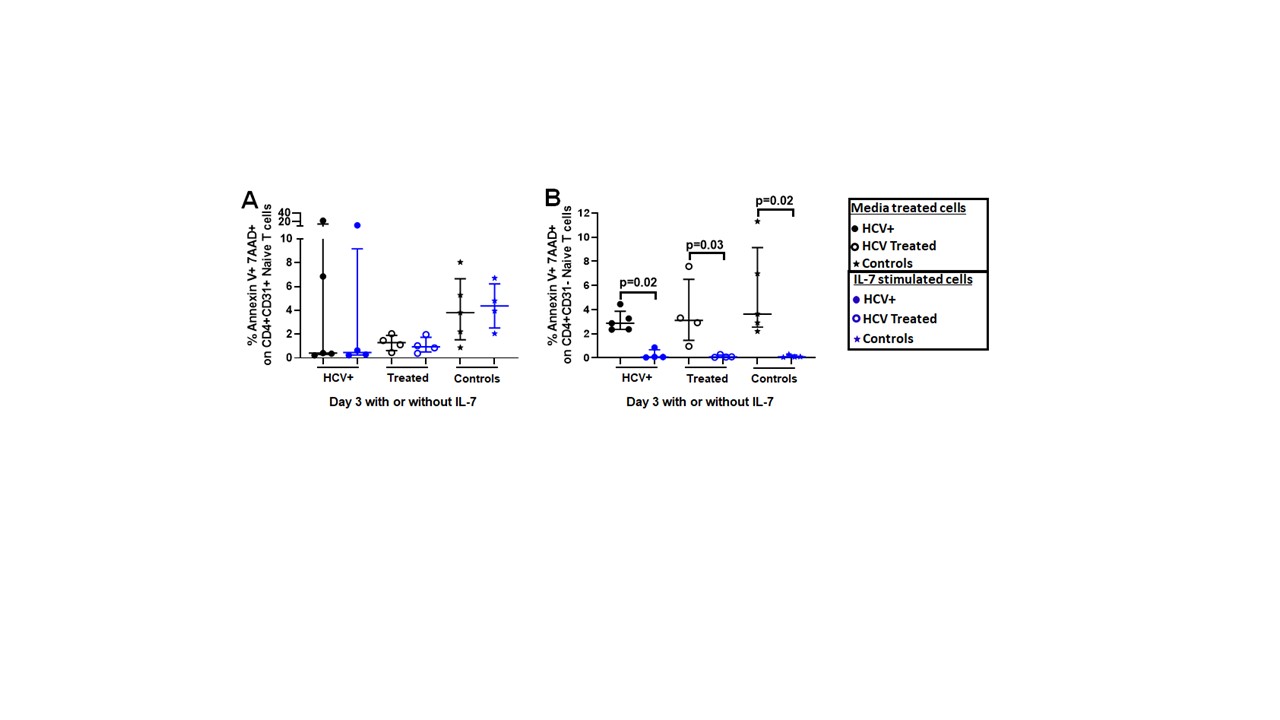

Supplement: Supplementary Figure 4 — The naïve CD4+CD31- T cells undergo spontaneous apoptosis at higher levels compared to the naïve CD4+CD31+ T cells before and after HCV DAA therapy and in absence of HCV infection. Magnetic bead purified (negative selection) naïve CD4 T cells from chronic HCV infected (filled circles, n=4), HCV DAA-treated (open circles, n=4) and age-range matched uninfected control (stars, n=5) groups were stimulated with or without 10ng/ml of recombinant human IL-7 for 3 days. On third day, flow cytometric analysis for apoptosis (AnnexinV and 7AAD) on naïve (CD27+CD45RA+) CD4+CD31+ (A) and CD4+CD31- (B) T cells was performed. Mann Whitney test was used for comparisons between two groups; p= <0.05 considered significant. [file Image_4.jpeg]

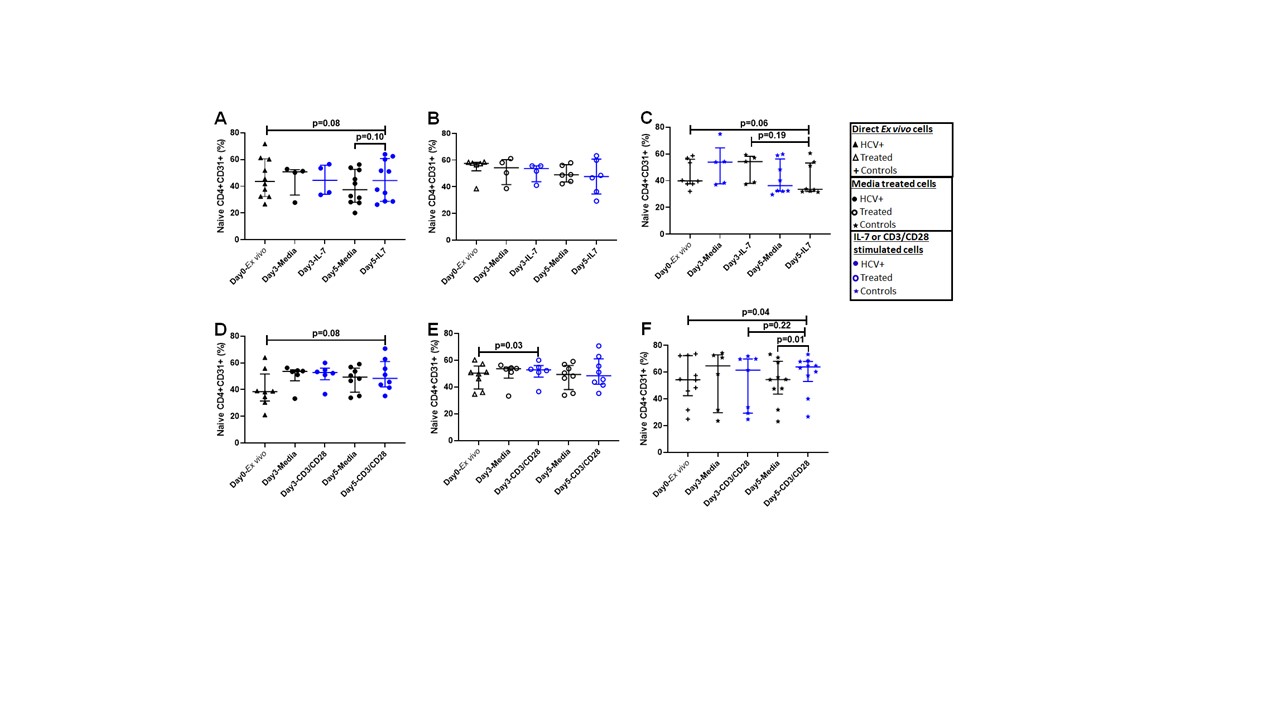

Supplement: Supplementary Figure 5 — CD31 expression on naïve CD4+ T cells is mostly stable during in vitro stimulation with recombinant IL-7 or CD3/CD28 activator in cells from HCV infected and DAA-treated persons. Magnetic bead purified (negative selection) naïve CD4 T cells from chronic HCV infected (filled circles, n=8; A, D), HCV DAA-treated (open circles, n=8; B, E) and age-range matched uninfected control (stars, n=7; C, F) groups were stimulated with 10ng/ml of recombinant human IL-7 (A–C) or 1ul anti-CD3/anti-CD28 Activator (D–F) for 5 days. On 0 (direct ex vivo), 3, and 5 days, flow cytometric analysis of CD31 expression on naïve (CD27+CD45RA+) CD4+ T cells was performed. Mann Whitney test was used for comparisons between two groups; p= <0.05 considered significant. [file Image_5.jpeg]
